# Targeting Membrane-Bound Viral RNA Synthesis Reveals Potent Inhibition of Diverse Coronaviruses Including the Middle East Respiratory Syndrome Virus

**DOI:** 10.1371/journal.ppat.1004166

**Published:** 2014-05-29

**Authors:** Anna Lundin, Ronald Dijkman, Tomas Bergström, Nina Kann, Beata Adamiak, Charles Hannoun, Eveline Kindler, Hulda R. Jónsdóttir, Doreen Muth, Joeri Kint, Maria Forlenza, Marcel A. Müller, Christian Drosten, Volker Thiel, Edward Trybala

**Affiliations:** 1 Department of Clinical Virology, University of Gothenburg, Göteborg, Sweden; 2 Institute of Immunobiology, Kantonal Hospital St.Gallen, St.Gallen, Switzerland; 3 Federal Department of Home Affairs, Institute of Virology and Immunology, Berne and Mittelhäusern, Switzerland; 4 Organic Chemistry, Department of Chemical and Biological Engineering, Chalmers University of Technology, Göteborg, Sweden; 5 Institute of Virology, University of Bonn Medical Centre, Bonn, Germany; 6 Department of Animal Sciences, Cell Biology and Immunology Group, Wageningen Institute of Animal Sciences, Wageningen University, Wageningen, The Netherlands; 7 Merck Animal Health, Bioprocess Technology & Support, Boxmeer, The Netherlands; 8 Vetsuisse Faculty, University of Berne, Berne, Switzerland; Johns Hopkins University - Bloomberg School of Public Health, United States of America

## Abstract

Coronaviruses raise serious concerns as emerging zoonotic viruses without specific antiviral drugs available. Here we screened a collection of 16671 diverse compounds for anti-human coronavirus 229E activity and identified an inhibitor, designated K22, that specifically targets membrane-bound coronaviral RNA synthesis. K22 exerts most potent antiviral activity after virus entry during an early step of the viral life cycle. Specifically, the formation of double membrane vesicles (DMVs), a hallmark of coronavirus replication, was greatly impaired upon K22 treatment accompanied by near-complete inhibition of viral RNA synthesis. K22-resistant viruses contained substitutions in non-structural protein 6 (nsp6), a membrane-spanning integral component of the viral replication complex implicated in DMV formation, corroborating that K22 targets membrane bound viral RNA synthesis. Besides K22 resistance, the nsp6 mutants induced a reduced number of DMVs, displayed decreased specific infectivity, while RNA synthesis was not affected. Importantly, K22 inhibits a broad range of coronaviruses, including Middle East respiratory syndrome coronavirus (MERS–CoV), and efficient inhibition was achieved in primary human epithelia cultures representing the entry port of human coronavirus infection. Collectively, this study proposes an evolutionary conserved step in the life cycle of positive-stranded RNA viruses, the recruitment of cellular membranes for viral replication, as vulnerable and, most importantly, druggable target for antiviral intervention. We expect this mode of action to serve as a paradigm for the development of potent antiviral drugs to combat many animal and human virus infections.

## Author Summary

Viruses that replicate in the host cell cytoplasm have evolved to employ host cell-derived membranes to compartmentalize genome replication and transcription. Specifically for positive-stranded RNA viruses, accumulating knowledge concerning the involvement, rearrangement and requirement of cellular membranes for RNA synthesis specify the establishment of the viral replicase complex at host cell-derived membranes as an evolutionary conserved and essential step in the early phase of the viral life cycle. Here we describe a small compound inhibitor of coronavirus replication that (i) specifically targets this membrane-bound RNA replication step and (ii) has broad antiviral activity against number of diverse coronaviruses including highly pathogenic SARS-CoV and MERS-CoV. Since resistance mutations appear in an integral membrane-spanning component of the coronavirus replicase complex, and since all positive stranded RNA viruses have very similar membrane-spanning or membrane-associated replicase components implicated in anchoring the viral replication complex to host cell-derived membranes, our data suggest that the membrane-bound replication step of the viral life cycle is a novel, vulnerable, and druggable target for antiviral intervention of a wide range of RNA virus infections.

## Introduction

Prior to the emergence of the highly pathogenic severe acute respiratory syndrome-associated coronavirus (SARS-CoV) in 2003 [1]–[3] only two circulating human coronaviruses (HCoVs), HCoV-229E [4] and HCoV-OC43 [5] causing relatively mild common cold-like respiratory tract infections, were known, and coronaviruses have not been regarded as significant threat for human health. Now, more than ten years later, the emergence of another highly pathogenic coronavirus of zoonotic origin, the Middle East respiratory syndrome coronavirus (MERS-CoV) [6]–[8], boosted community awareness towards the pending need to develop effective therapeutic options to combat coronavirus infections.

Coronaviruses are enveloped viruses and their positive strand RNA genome, the largest of all RNA viruses, encodes for as many as 16 non-structural proteins (nsps), 4 major structural proteins, and up to 8 accessory proteins (reviewed in [9]). Many of these proteins provide essential, frequently enzymatic, functions during the viral life cycle and are therefore attractive targets for antiviral intervention. Antiviral strategies are mainly proposed for targeting coronavirus entry and essential enzymatic functions, such as coronavirus protease or RNA-dependent RNA polymerase (RdRp) activities. For example, the spike (S) protein mediates binding of different HCoVs to their specific cellular receptors [10]–[14], an event associated with preferential virus tropism for either ciliated or non-ciliated cells of the airway epithelium [15]. The S protein also mediates fusion between lipids of the viral envelope and the host cell plasma membrane or membranes of endocytic vesicles to promote delivery of viral genomic RNA into the cytoplasm. Virus binding and cell entry events can be inhibited by antibodies directed against the S protein, antibodies or small molecules interfering with the virus receptors, or synthetic peptides derived from the fusion-triggering heptad repeat regions of the S protein (reviewed in [16]). Following virus entry, the coronavirus genome, a positive sense, capped and polyadenylated RNA strand, is directly translated resulting in the synthesis of coronavirus replicase gene-encoded nsps. Coronavirus nsps are translated as two large polyproteins harboring proteolytic enzymes, namely papain-like and chymotrypsin-like proteinases that extensively process coronavirus polyproteins to liberate up to 16 nsps (nsp 1–16) [9], [17]–[20]. These proteolytic functions are considered essential for coronavirus replication and, consequently, a number of candidate drugs were reported to inhibit coronavirus polyprotein processing [21]–[26]. Likewise, the coronavirus RdRp activities, which reside in nsp8 [27] and nsp12 [28], are considered essential for coronavirus replication and attractive targets for antiviral intervention. In addition to these classical drug targets, coronaviruses encode an array of RNA-processing enzymes representing additional candidate targets. These include a helicase activity linked to an NTPase activity in nsp13, a 3′-5′-exonuclease activity linked to a N7-methyltransferase activity in nsp14, an endonuclease activity in nsp15, and a 2′-O-methyltransferase activity in nsp16 (reviewed in [28]).

Like all positive strand RNA viruses, coronaviruses synthesize viral RNA at organelle-like structures in order to compartmentalize this critical step of the viral life cycle to a specialized environment that is enriched in replicative viral and host-cell factors, and at the same time protected from antiviral host defense mechanisms [29]–[31]. There is now a growing body of knowledge concerning the involvement, rearrangement and requirement of cellular membranes for RNA synthesis of a number of positive-strand RNA viruses, including coronaviruses [30], [32]–[35]. Three coronaviral nsps, i.e., nsp3, nsp4, and nsp6 [9], [36], [37] are thought to participate in formation of these sites for viral RNA synthesis. In particular, these proteins contain multiple trans-membrane domains that are thought to anchor the coronavirus replication complex through recruitment of intracellular membranes to form a reticulovesicular network (RVN) of modified, frequently paired, membranes that includes convoluted membranes [32] and double membrane vesicles (DVM) [38] interconnected via the outer membrane with the rough ER [32]. Indeed, Angelini and colleagues [39] have recently shown that co-expression of all three transmembrane domain-containing SARS-CoV nsps (nsp3, nsp4, and nsp6) is required to induce DMVs that are similar to those observed in SARS-CoV-infected cells. Such organelle-like compartments harboring membrane-bound replication complexes show remarkable parallels amongst a broad range of positive-strand RNA virus families, and are potentially evolutionary linked to similar mechanisms in the life cycle of double-strand (ds)RNA, reverse-transcribing, and cytoplasmic replicating DNA viruses [29]. Coronavirus ER-derived DMVs are induced early after virus entry into the host cell cytoplasm [9], [32], [34], [38]–[43], and display striking similarities to DMVs induced by hepatitis C virus [33]. The evolutionary conservation of engaging host cell-derived organelle-like membranous structures for virus RNA synthesis and genetic evidence that impairment of coronavirus DMV integrity is associated with severe reduction of virus replication [44], [45] suggest that antiviral intervention by targeting membranes involved in virus replication represents an attractive, however yet largely unexplored approach.

In this work, we describe a novel inhibitor of coronavirus replication that specifically interferes with membrane-bound coronaviral RNA synthesis. This novel mode-of-action is characterized by severe impairment of DMV formation that results in near-complete inhibition of RNA synthesis. Notably, the inhibitor displayed antiviral activity against a broad range of animal and human coronaviruses, including the recently emerging MERS-CoV.

## Results

### Identification of anti-HCoV-229E hit compound K22

**Figure 1:**
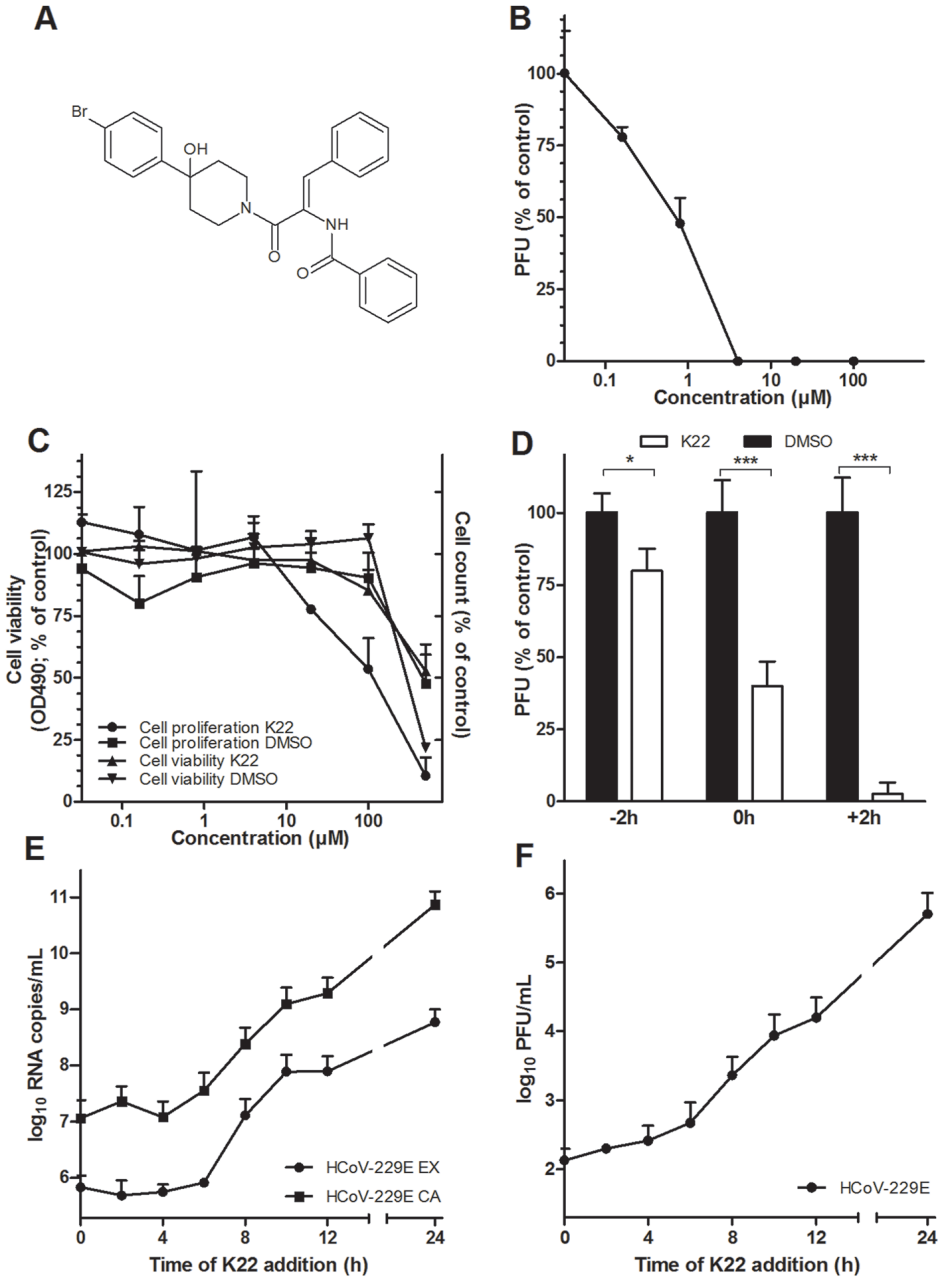
K22 structure, antiviral activity, and cytotoxicity. (**A**) K22 structure. (**B**) Anti-HCoV-229E activity of K22 in MRC-5 cells. K22 and HCoV-229E were added to MRC-5 cells, and the number of viral plaques developed after 48 h were assessed. Data shown are means (±SD) of duplicate determinations from three independent experiments. PFU, plaque forming unit. (**C**) Viability and proliferation of MRC-5 cells in the presence of K22. MRC-5 cells were incubated with K22 or DMSO solvent for 48 h at 37°C and the cell viability determined using tetrazolium-based reagent while cell proliferation was assayed by counting of cells. Data shown are means (±SD) of duplicate determinations from two independent experiments. (**D**) K22 affects the post-entry phase of viral life cycle. K22 (4 µM) or DMSO solvent were incubated with cells for a period of 2 h either before (−2 h), during (0 h) or after a 2 h period of cell inoculation with HCoV-229E, and the number of viral plaques developed after 48 h were assessed. Data shown are means of duplicate determinations from three independent experiments.*P<0.05; *n* = 3. ***P<0.005; *n* = 3. (**E-F**) K22 exhibits potent antiviral activity when added up to 6 h after infection of cells. MRC-5 cells were inoculated with HCoV-229E at a moi of 0.05 for 45 min at 4°C, and K22 (10 µM) added at specific time points relative to the end of inoculation period. The culture medium and cells were harvested after 24 h of incubation at 37°C, and the viral RNA (**E**) and infectivity (**F**) determined. Data shown are means (±SD) of duplicate determinations from two independent experiments. EX, extracellular medium; CA, cell-associated sample.

To identify novel inhibitors of coronavirus infectivity we screened the ChemBioNet collection of 16671 compounds for antiviral activity against HCoV-229E. To this end, MRC-5 cells growing on 384-well plates were supplemented with a specific library compound (20 µM) and then inoculated with HCoV-229E. Compounds that reduced or abolished viral cytopathic effect were re-tested in 24-well plate format for more precise evaluation of their antiviral potential. This two-step screening procedure resulted in several hits including two structurally similar compounds referred to as K22 (Figure 1A) and J15 (Figure S1A). The former compound, K22, whose structural name is (*Z*)-N-(3-(4-(4-bromophenyl)-4-hydroxypiperidin-1-yl)-3-oxo-1-phenylprop-1-en-2-yl)benzamide was examined in detail. The compound was completely soluble in medium up to 50 µM. The concentration of K22 that inhibited the number of HCoV-229E plaques by 50% (IC_50_) was 0.7 µM (Figure 1B). K22 did not reduce viability of MRC-5 cells by >50% (CC_50_) at a concentration range of 0.032–500 µM (Figure 1C). However this compound decreased proliferation of MRC-5 cells with a CC_50_ value of 110 µM (Figure 1C). Hence, using the CC_50_ value determined in cell proliferation assay, the selective index for K22, i.e. the CC_50_/IC_50_ quotient, was 157. Compound J15, although showing anti-HCoV-229E activity similar to that of K22 exhibited a somewhat less favorable cytotoxicity profile in the cell viability assay (Figure S1B).

### K22 inhibits HCoV-229E during the early, post entry phase of the viral life cycle

To assess which step of the HCoV-229E life cycle is affected by K22, a time-of-addition/removal experiment was performed. K22 (4 µM) was incubated with cells for a period of only two hours either prior to, during, or after infection with HCoV-229E. As shown in Figure 1D, K22 treatment prior to infection resulted in only marginal reduction of virus infectivity thus excluding blockade of cellular receptor(s) for HCoV-229E as its mode-of-action. Simultaneous addition of K22 and virus resulted in ∼50% reduction of virus infectivity suggesting that the compound may interact with viral particles thus inactivating their binding or cell-entry activity. To clarify this possibility, the virus was incubated with ∼70 IC_50_ doses of K22 or DMSO solvent for 15 min at 37°C, followed by virus dilution and its titration at non-inhibitory compound concentrations. Similar titers were observed for the virus treated with K22 (7.2×10^5^/ml±8.9%) and DMSO (7.5×10^5^/ml±4.7%) (n = 2; two experiments), indicating that K22 exhibited no virus particle-inactivating activity. Thus, the ∼50% reduction in plaque number (Figure 1D) observed by simultaneous addition of K22 and virus is likely due to cellular uptake of K22 and inhibitory activity of probably not yet metabolically processed compound during a very early step of virus replication rather than the drug binding to viral particles and interference with their penetration into cells. This idea is further corroborated by the most pronounced inhibition of HCoV-229E replication when K22 was added after infection (Figure 1D). To more precisely determine the time window of efficient K22-mediated inhibition of HCoV-229E, K22 (10 µM) was added to infected cells at different time points post infection (p.i.), and intra- and extracellular viral RNA, and infectious particles were quantified at 24 hours p.i.. As shown in Figures 1E-F, K22 addition within the first 6 hours p.i. resulted in near complete inhibition of viral RNA synthesis and ∼1000-fold reduction of produced infectious virus, suggesting that K22 inhibits most potently post virus entry during the early phase of the HCoV-229E life cycle.

### K22 resistant mutants contain substitutions in nsp6

**Figure 2:**
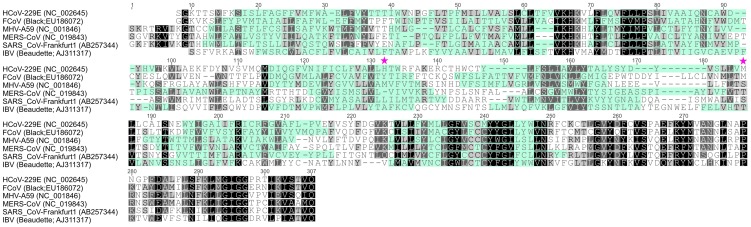
Alignment of coronavirus nsp6 sequences. Alignment of nsp6 sequences derived from coronaviruses used in this study was performed with Geneious Software (Biomatters Ltd, New Zealand). Coronavirus species and corresponding GenBank accession numbers are indicated. Membrane domains predicted by TMHMM Server v. 2.0 (http://www.cbs.dtu.dk/services/TMHMM/) are indicated by cyan shading while conserved amino acid residues are highlighted by black/grey shading. K22 resistance-conferring mutations in HCoV-229E nsp6, identified in this study, are depicted.

**Figure 3:**
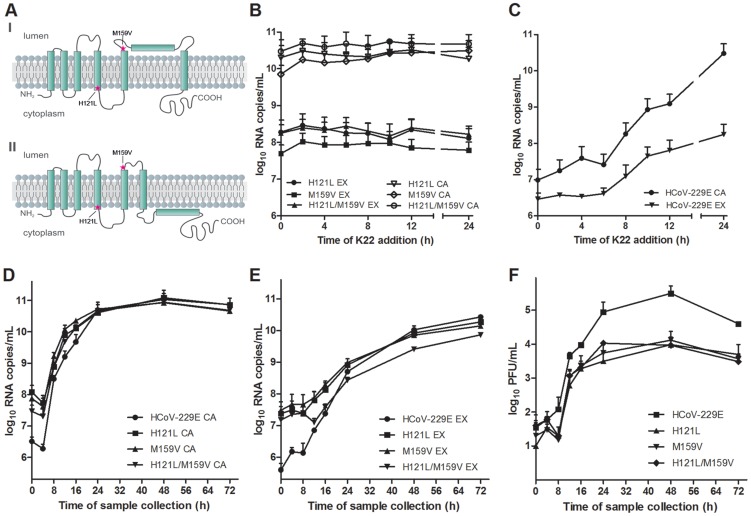
Analysis of recombinant HCoV-229E nsp6 mutants. (**A**) Predicted topological structure of HCoV-229E nsp6 indicating the location of K22 resistance mutations. Concerning transmembrane domains VI and VII two proposed topologies are shown. (**B-C**) Recombinant nsp6 mutant viruses are resistant to K22. MRC-5 cells were inoculated with nsp6 recombinant HCoV-229E^H121L^, HCoV-229E^M159V^, HCoV-229E^H121L/M159V^ or wild-type HCoV-229E at a moi of 0.05 for 45 min at 4°C, and K22 (10 µM) was added at specific time points relative to the end of inoculation period. The infectious cell culture medium and cells were harvested after 24 h of incubation at 37°C, and copy numbers of cell-associated (CA) or extracellular (EX) viral RNA was determined. Data shown are means (±SD) of duplicate determinations from two independent experiments. (**D-F**) Replication kinetics of recombinant nsp6 mutant viruses. MRC-5 cells were inoculated with nsp6 recombinant HCoV-229E^H121L^, HCoV-229E^M159V^, HCoV-229E^H121L/M159V^ or wild-type HCoV-229E at an moi of 0.05 for 1 h at 4°C. The infectious cell culture medium and cells were harvested at specific time points relative to the end of inoculation period, and copy numbers of cell-associated (CA; **D**) or extracellular (EX; **E**) viral RNA and infectivity (**F**) was determined. Data shown are means (±SD) of duplicate determinations from two independent experiments.

To obtain further insight concerning the target of K22 inhibition we aimed to generate K22-resistant mutants and therefore subjected plaque purified HCoV-229E to 10–13 consecutive passages on MRC-5 cells in presence of increasing concentrations of K22 (2–16 µM). In two independent experiments we isolated and plaque purified several variants displaying moderate (∼2-fold) to strong (∼12-fold) K22 resistance (IC_50_ of 1.6–8.5 µM; Table 1). Whole genome sequencing analysis of wild type (wt) HCoV-229E, mock passaged virus, and K22 passaged virus revealed two amino acid substitutions within nsp 6 (H121L; M159V) that were associated with strong K22 resistance (Table 1). Sequence alignment and prediction of potential transmembrane regions of nsp6 homologs of HCoV-229E and other coronaviruses used in this study, revealed presence of 7 potential membrane-spanning domains (Figure 2) 6 of which are proposed to be used as membrane anchors in other coronaviruses [36], [37], and that mutations conferring resistance to K22 are located in or near these regions (Figure 3A). Subsequent generation of recombinant mutants, designated HCoV-229E^H121L^, HCoV-229E^M159V^, and HCoV-229E^H121L/M159V^, carrying the nsp6 mutations individually or combined by reverse genetics confirmed that these mutations confer resistance to K22 inhibition as revealed by plaque inhibition (Table 1) and the time-of-addition (Figures 3B-C) assays. Thus, as expected from the previous experiment (Figure 1E), K22 addition within the first 6 hours p.i. with the wt HCoV-229E resulted in near complete inhibition of viral RNA synthesis (Figure 3C), an effect completely abrogated in the drug-resistant recombinant mutant viruses (Figure 3B). Notably, although the amount of intracellular (Figure 3D) and extracellular (Figure 3E) viral RNA was comparable between K22-resistant mutants and parental wt HCoV-229E, production of infectious particles during infection with K22-resistant mutant viruses was greatly reduced (up to 34 fold at 48h p.i.) (Figure 3F). This difference cannot be attributed to the presence of free viral RNA in preparations of extracellular virus, since the treatment of K22-resistant HCoV-229E^M159V^ mutant virus with ribonuclease A did not reduce the quantity of viral RNA (Figure S2). This observation suggests that K22 resistance-conferring mutations in nsp6 are associated with a fitness cost (reduced specific infectivity).

**Table 1:** Alterations detected in the K22 resistant variants of HCoV-229E.

	Alteration^a^			
Viral variant	Nucleotide	Amino acid (protein)	K22 sensitivity	GenBank accession no.
Initial^b^	None	None	0.7^c^	KF293664
K22 passage 10	a10455t	H121L (Nsp6)	9.8 (14)^d^	KF293666
	c19463t	T281I (Nsp15)		
	c26667t	P328S (Nucleocapsid)		
A	a10455t	H121L (Nsp6)	8.2 (12)	KF285470
B	a10455t	H121L (Nsp6)	8.2 (12)	KF285471
D	a10455t	H121L (Nsp6)	7.6 (11)	KF285472
G	a10455t	H121L (Nsp6)	6.9 (10)	KF285473
K	c19463t	T281I (Nsp15)	1.6 (2)	KF285481
	c26667t	P328S (Nucleocapsid)		KF293662
L	c19463t	T281I (Nsp15)	2.2 (3)	KF285482
	c26667t	P328S (Nucleocapsid)		KF293663
K22 passage 13 - M^e^	a10568g	M159V (Nsp6)	6.7 (10)	KF285474
	a23130c	N854T (Spike)		KF285480
N	a10568g	M159V (Nsp6)	7.1 (10)	KF285475
O	a10568g	M159V (Nsp6)	7.7 (11)	KF285476
P	a10568g	M159V (Nsp6)	8.5 (12)	KF285477
Q	a10568g	M159V (Nsp6)	7.7 (11)	KF285478
R	a10568g	M159V (Nsp6)	6.8 (10)	KF285479
HCoV-229E^f^			0.6	
gHCoV-229E^H121L^	a10455t	H121L (Nsp6)	7.2 (12)	
gHCoV-229E^M159V^	a10568g	M159V (Nsp6)	6.3 (11)	
gHCoV229E^H121L/M159V^	a10455t	H121L (Nsp6)	8.2 (14)	
	a10568g	M159V (Nsp6)		

a Detected by comparison of the nucleotide sequences of HCoV-229E subjected to 10–13 passages in the presence of K22 including its plaque purified variants A-R with those of initial virus or mock-passaged virus (accession number KF293665).

b Plaque purified HCoV-229E that served as initial material for the virus passages.

c IC50 (µM).

d Fold resistance to K22 as related to initial virus is shown in parentheses.

e Virus preparation and its plaque purified variants M-R obtained in separate K22 selection experiment.

f The virus used for preparation of recombinant nsp6 mutants.

g K22 resistant recombinant viruses.

### K22 treatment results in loss of DMVs

**Figure 4:**
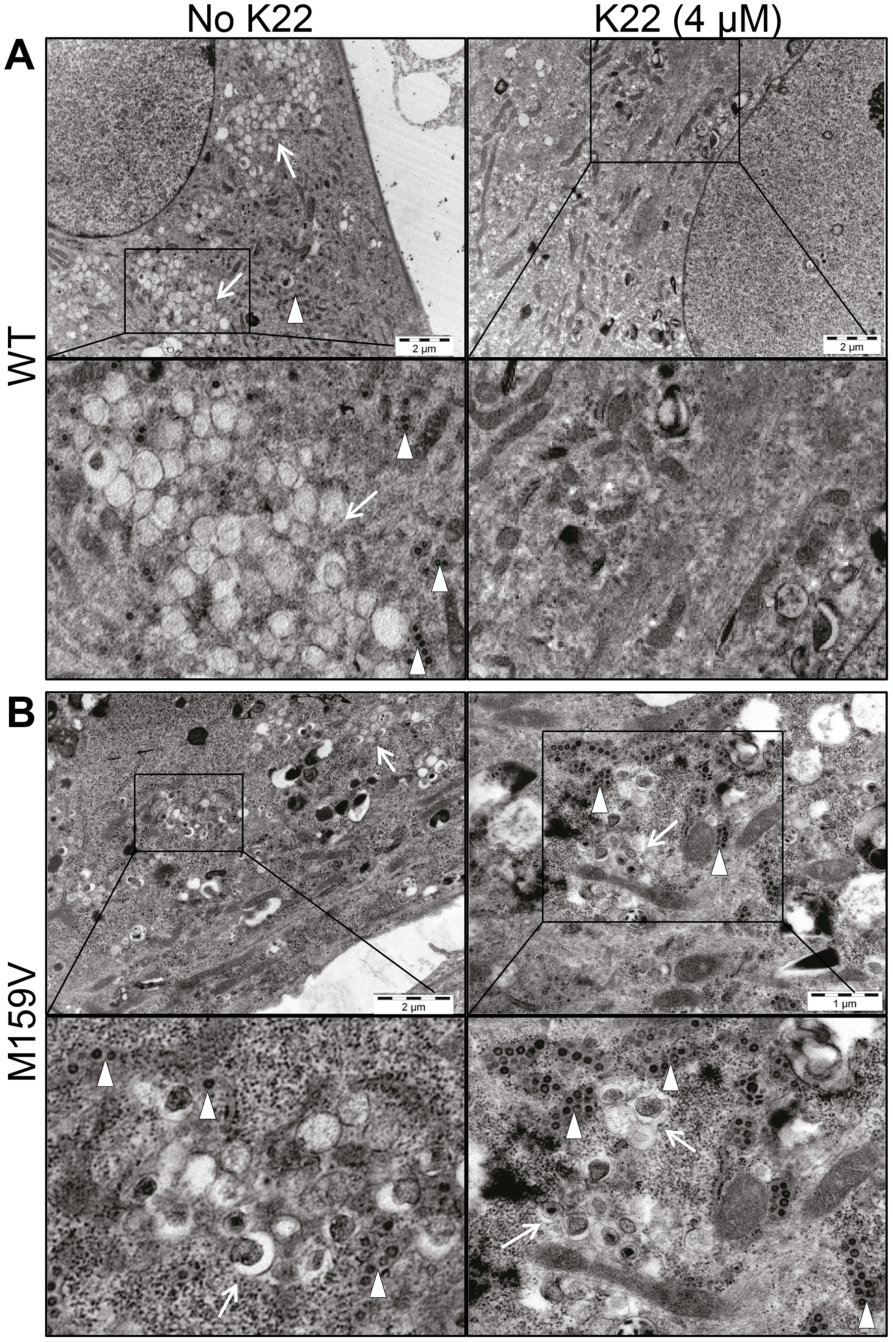
K22 affects formation of double membrane vesicles (DMVs). MRC-5 cells growing on Melinex polyester film were infected with wild type HCoV-229E (WT) or with K22-resistant recombinant nsp6 mutant HCoV-229E^M159V^ (M159V) and incubated for 18 h at 37°C with or without K22. The cells were then fixed with glutaraldehyde and processed for electron microscopy without their scrapping or pelleting. (**A**) Electron micrographs of cells infected with WT virus show presence of perinuclear clusters of DMVs (arrow) and viral particles (arrowhead), and the lack of their production upon K22 treatment (4 µM). (**B**) Note presence of DMVs and viral particles in cells infected with K22-resistant nsp6 recombinant HCoV-229E^M159V^ (M159V) irrespective of the addition of K22. Each image shown was selected from a pool of over 30 images captured in three separate experiments.

**Figure 5:**
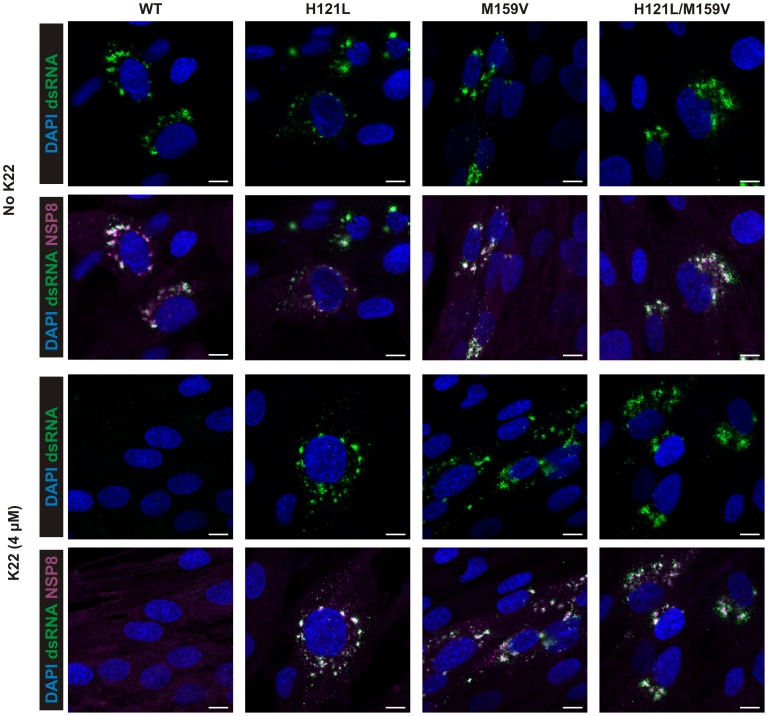
K22 affects formation of coronavirus replication complex in cells. MRC-5 cells were infected with wild type HCoV-229E (WT) and K22-resistant recombinants HCoV-229E^H121L^ (H121L), HCoV-229E^M159V^ (M159V), and HCoV-229E^H121L/M159V^ (H121L/M159V) and incubated for 18 h with or without the presence of K22. The cells were then fixed with 4% paraformaldehyde and immunostained for immunofluorescence analysis. Note the lack of detection of dsRNA and nsp8 upon K22 treatment (4 µM) of cells infected with WT but not recombinant viruses. Scale bar is 10 µM.

The observation that amino acid substitutions in nsp6 confer K22 resistance strongly suggests a mode-of-action based on interference with host cell membranes required for coronavirus replication. Nsp6 is expressed as a membrane-spanning integral component of the viral replication complex, and is, together with nsp3 and nsp4, implicated in anchoring the coronavirus replicase complex to DMVs or related membrane structures [9], [36], [37], [39], [43]. Indeed, there is genetic and experimental evidence concerning nsp4-mediated alterations of coronavirus DMVs [44], [45], and that ectopic expression of nsp6 results in the formation of ER-derived vesicles [46]. We therefore assessed if K22 may impact the formation of coronavirus-induced DMV by electron microscopy (Figure 4). As expected, perinuclear DMV clusters as well as viral particles were readily detectable in wt HCoV-229E-infected cells (Figure 4A). In sharp contrast, no DMV clusters or viral particles were detectable in wt HCoV-229E-infected and K22-treated (4 µM) cells (Figure 4A). Since double-stranded (ds) RNA is indicative of coronavirus replication and has been shown to reside predominantly within the inner lumen of coronavirus-induced DMVs [32] we also performed immunofluorescence analysis and stained HCoV-229E-infected cells for viral replicase complex (nsp8) and dsRNA. Strikingly, the characteristic perinuclear immunofluorescence staining pattern for viral replicase complexes and dsRNA visible in wt HCoV-229E-infected cells was completely absent under K22 treatment (Figure 5), confirming the remarkable efficacy of K22-mediated inhibition of viral replication and supporting the notion that K22 blocks the formation of DMVs. In contrast to parental wt HCoV-229E and irrespectively whether K22 was applied, recombinant K22 escape mutants were still capable of inducing the formation of DMVs (Figure 4B) and displayed the characteristic staining pattern for replicase complexes and dsRNA (Figure 5). Likewise, compound J15 efficiently blocked replication (Figure S1B) and DMV formation of wt HCoV-229E but not K22 resistant nsp6 recombinant HCoV-229E^M159V^ (Figure S3) suggesting that J15 may have the same target and mode-of-action. Notably, in cells infected with K22 escape mutants the overall number of DMVs per cell was reduced (30.3±29.7 in HCoV-229E^M159V^ versus 65±50.1 in wt HCoV-229E infected cells; *P*<0.05; *n* = 20), similar as previously described for mouse hepatitis virus (MHV) nsp4 mutants [44], [45], while the number of intracellular viral particles that were often packed in tubular vesicle-like structures (Figures 4A-B) was comparable to that of wt virus (471.8±212.6 in HCoV-229E^M159V^ versus 438.3±96.8 in wt virus infected cells; *n* = 10). We could also frequently detect DMVs displaying partially collapsed inner membranes in cells infected with K22 escape mutants (irrespectively whether or not K22 was applied; Figure 4B), again similarly as reported for MHV nsp4 mutants [45], suggesting that nsp6, like nsp4, has a pivotal role in coronavirus DMV formation. Overall, these findings demonstrate that the antiviral activity of K22 (and that of the structurally similar compound J15) results in complete loss of DMVs. This efficient block in replication can be overcome by resistance mutations in nsp6, and DMVs induced by nsp6 mutant viruses are reduced in numbers and structurally impaired – both findings concurring with the established function of nsp6 in DMV formation.

### K22 does not impact cellular autophagy

Our data show that K22 targets a very early step in the HCoV-229E life cycle, and the appearance of resistance-conferring mutations in nsp6 suggests that K22 impairs DMV formation. We therefore assessed if K22 treatment may, independent of virus infection, impact autophagy, a cellular process displaying similarities to coronaviral DMV formation. To this end we first transfected Huh7 cells with a plasmid encoding LC3B-GFP in order to trace rapamycin-induced autophagsomes by life imaging. This analysis revealed that three to six hours after adding rapamycin to the culture medium green fluorescent autophagocytic vesicles become apparent, irrespectively if K22 (20 µM) was added or not (data not shown). We corroborated this result by immunofluorescence analysis of Huh7 cells that were stained for endogenous LC3B at six hours post addition of rapamycin. As shown in supplementary Figure S4 rapamycin-incuced autophagocytic vesicles were again readily detectable in the presence of K22 (20 µM), suggesting that K22 does not impact cellular autophagy.

### K22 inhibits a number of diverse coronaviruses

**Figure 6:**
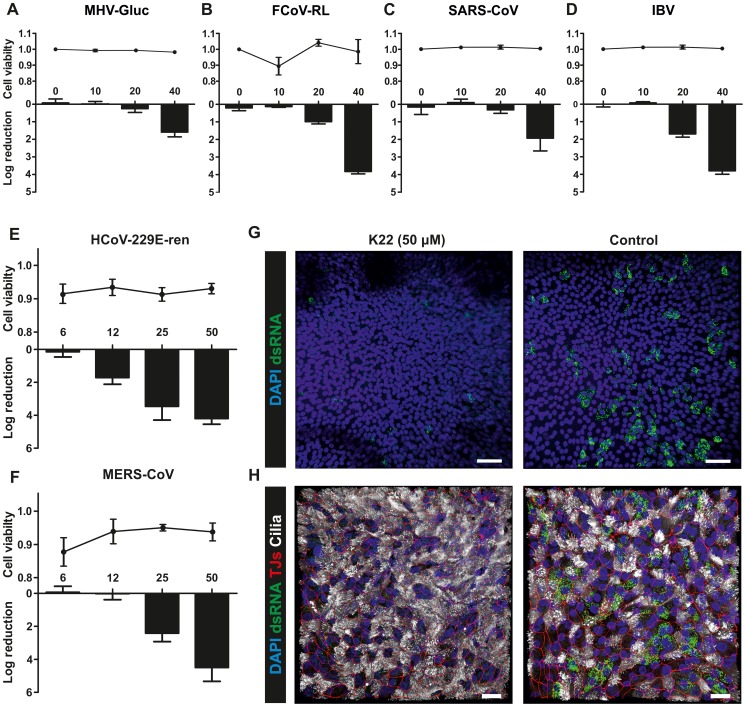
K22 affects replication of diverse coronaviruses including MERS-CoV. (**A-D**) The log reduction of the antiviral activity (bars) and cell toxicity ratio (data points above bars) of K22 during MHV-Gluc (**A**), FCoV-RL (**B**), SARS-CoV (**C**) and IBV (**D**) infection on representative continuous cell lines of murine (L-929 cells; **A**), feline (FCWF cells; **B**), or primate (Vero cells; **C-D**) origin. Data are shown as mean (±SD) of a representative experiment, from two independent experiments performed in triplicate. Toxicity values for Vero cells in panels C and D are derived from the same experiments. (**E-F**). The log reduction of the antiviral activity (bars) and cell toxicity ratio (data points above bars) of K22 in HCoV-229E-ren (**E**) and MERS-CoV (**F**) infected differentiated human airway epithelial (HAE) cultures. Data are shown as mean (±SD) of three independent experiments performed in triplicate (log reduction), or mean (±SD) of a representative experiment, from two independent experiments performed in triplicate (cell viability). (**G-H**) Immunofluorescence analysis of HAE cultures infected with MERS-CoV in presence or absence of K22 in a representative overview (**G**, 20x; **H**, 40x) confocal Z-stack image. Stainings were performed using antibodies directed against (**G**) dsRNA (green), and DAPI (cell nucleus; blue), and (**H**) dsRNA, DAPI, β-tubulin (ciliated cells; white), and ZO1 (tight junctions, red). Scale bars are 50 (**G**) or 20 (**H**) µm.

Since K22 inhibits a crucial step in the HCoV-229E life cycle, we assessed the antiviral activity of K22 against a panel of diverse coronaviruses representing the major phylogenetic lineages of α-, β- and ???-coronaviruses. As shown in Figure 6A-D and supplementary Figure S5, K22 indeed displayed antiviral activity against recombinant MHV (strain A59 [47]) expressing *Gaussia* luciferase as marker for virus replication, recombinant type-I feline coronavirus (FCoV; strain Black [48]) expressing *Renilla* luciferase as marker for virus replication, avian infectious bronchitis virus (IBV; strain Beaudette [49]), and SARS- CoV (strain Frankfurt-1 [50]), suggesting that K22 targets a broad range of coronaviruses. Furthermore, there was no cytotoxicity detectable in cells of feline (FCWF cells), murine (L929 cells), and primate (Vero cells) origin in the K22 concentration range assessed, and analysis of K22 cytostatic activities in the cell proliferation assay revealed CC_50_ values ≥40 µM (Table S1), i.e., the highest drug concentration used in antiviral assays. Notably, the efficacy of K22-mediated inhibition varied amongst different coronaviruses, however whether this is related, as in HCoV-229E, to nsp6 function would require generation and analysis of K22 resistant variants for all coronaviruses tested. In contrast, K22 exhibited little or no effect on replication of poliovirus (Figure S6), a pathogen that like coronaviruses induces rearrangement of cellular membranes to assist RNA replication.

### Inhibition of HCoV-229E and MERS-CoV in primary human airway epithelia cultures

Finally, we assessed the efficacy of K22 inhibition in the primary target cells of respiratory virus infection, the human airway epithelium. Fully differentiated primary human airway epithelia (HAE) cultures [15], [51] derived from three different donors and grown under air-liquid interphase conditions were infected with a recombinant HCoV-229E expressing *Renilla* luciferase as marker for virus replication [52], and with MERS-CoV [8], [51]. MERS-CoV was first described in 2012 and was isolated from a 60-year old man with acute pneumonia, renal failure and fatal outcome in Saudi Arabia [8]. The virus is most likely of zoonotic origin [7], [53] and by February 2014 the number of laboratory-confirmed cases of MERS-CoV infection reported to the World Health Organization exceeded 182, including more than 79 cases with fatal outcome. We have previously shown that MERS-CoV can readily replicate on primary HAE cells [51] by infecting non-ciliated cells expressing the cellular receptor dipeptidyl peptidase 4 [14]. As shown in Figure 6, HCoV-229E and MERS-CoV infections were inhibited by K22 treatment with remarkable efficacy, illustrated by reduction of viral replication by several orders of magnitude (Figure 6E-F) and substantial reduction of dsRNA in MERS-CoV-infected primary HAE cultures (Figure 6G-H). This result demonstrates that the broad anti-coronaviral activity of K22 makes this compound particularly promising for the development of efficacious treatment options for emerging coronaviruses, such as MERS-CoV.

## Discussion

Here we describe the discovery of a novel class of inhibitor and propose a mode-of-action that targets membrane-bound viral replication. Like all positive strand RNA viruses, coronaviruses employ host cell membranes to assemble the viral replicase complex. This evolutionary conserved strategy provides a compartment for viral RNA synthesis that is enriched in replicative viral and host cell-derived proteins and believed to protect from antiviral host cell defense mechanisms. The remarkable efficacy of K22-mediated inhibition of coronavirus replication confirms that the employment of host cell membranes for viral RNA synthesis is a crucial step in the coronavirus life cycle, and importantly, demonstrates that this step is extremely vulnerable and also druggable for antiviral intervention.

The observation that K22 resistance is mediated through mutations in nsp6 defines transmembrane domain-containing nsps implicated in anchoring viral replicase complexes to host cell-derived membranes, as novel targets for anti-coronaviral intervention. Moreover, we expect this mode-of-action to serve as a paradigm for the development of similar antiviral drugs to combat infections caused by many other positive strand RNA viruses. Notably, resistance conferring mutations in nsp6 emerged only after 10–13 consecutive passages of HCoV-229E under K22 selection, and we were so far not successful in obtaining K22-resistant MHV-A59 mutants (data not shown). This suggests that escape mutations in membrane domain-containing coronavirus nsps compatible with maintaining efficient RNA synthesis are limited. In addition, the nsp6 escape mutants we have obtained for HCoV-229E display a remarkable reduction of specific infectivity. Thus, although RNA synthesis appears to be unaffected and viral RNA detected in preparations of extracellular virus was ribonuclease insensitive implying its adequate package in viral particles, mutations in nsp6 seem to reduce virus fitness. Thus, it is conceivable that the nsp6 mutants may be functionally impaired during an early step in the viral life cycle. Since dsRNA is localized in DMVs and nsp6 escape mutants induced decreased number of DMVs that are structurally impaired, it is possible that the reduced specific infectivity of these viruses could be related to dsRNA-triggered innate immune responses.

SARS-CoV nsp6 was recently found to contribute to the establishment of the virus-induced RVN by promoting vesicle formation in transfected cells [39], and our observation that K22 resistant mutants generated decreased number of DMVs implies that specific alterations may adversely affect the vesicle-forming capability of nsp6. Nsp6 of HCoV-229E (this report), MHV, and SARS-CoV [36], [37] is predicted as a hexaspaning protein comprising a conserved C-terminal cytoplasmic tail. The latter domain may serve as a wedge-like amphipathic helix which upon insertion into the lipid membrane can trigger its bending due to induction of positive membrane curvature (reviewed in [54]). The vesicle formation would also require a putative ion channel activity that depolarizes curved membranes thus facilitating their fusion and vesicle scission. The question as to whether nsp6 or other components of the coronavirus replicase complex exhibit such activities would require further investigation.

Although our data reveal that the K22 escape mutations occur in nsp6, further binding experiments are required to clarify whether K22 targets nsp6 directly. We observed that K22 is most active in inhibiting replication of the tested α-coronaviruses (HCoV-229E, FCoV) and the γ-coronavirus IBV, whereas amongst β-coronaviruses K22 was highly active in inhibiting MERS-CoV, but only moderately against MHV or SARS-CoV (Figure 6). It is conceivable that K22 may strong inhibit α-coronaviruses, since K22 has been identified by screening for anti-HCoV-229E activity. However, the limited nsp6 sequence similarity between coronaviruses (Figure 2) does not allow predicting the strength of K22-mediated inhibition of replication based on nsp6 homology. We also like to address in future studies a question of how the moderately resistant virus variant L (containing mutations in nsp15 and nucleocapsid) can escape K22-mediated inhibition of replication. This variant, in contrast to these containing resistance mutations in nsp6, exhibited only moderate resistance to K22 (∼2-3-fold) and was not consistently selected in separate selection experiments. Although nsp15 and nucleocapsid protein have not yet been described as being directly involved in DMV formation, these proteins are components of the replicase complex that may somehow affect/modulate nsp6 functions, and compensatory mutations in these proteins may partially relieve K22 blockade of nsp6. An alternative possibility is that the actual K22 target may be a cellular protein or a process of recruitment of a cellular protein that participates in coronavirus-induced membrane rearrangements by interacting with nsp6. While we could not observe any detectable impact of K22 on the formation of autophagosomes, further studies are required to address if K22 may target similar vesicles, such as EDEMosomes [41]. Both possibilities are compatible with the observed phenotype of DMV impairment and the detection of resistance mutations at regions of HCoV-229E nsp6 that are structurally conserved while displaying only limited sequence similarity. It is thus conceivable that membrane domain-containing nsp3 and nsp4 may represent additional drug targets. Similar as described for the related arteriviruses, where co-expression of membrane-spanning nsp2 and nsp3 results in membrane alterations and DMV formation similar to those observed during arterivirus infection [55], [56], co-expression of coronavirus nsp3, nsp4 and nsp6 is required to produce coronavirus-like membrane rearrangements including DMVs [39]. Expression of nsp3, nsp4 or nsp6 alone or in combinations of two induces aberrant membrane rearrangements that only partially mimic membrane structures known from coronavirus infection [39]. Thus, there is growing evidence that nsp3, nsp4, nsp6, and possibly ER membrane-resident host cell proteins [41], [57], orchestrate critical events that lead to the development of suitable membrane structures facilitating coronavirus RNA synthesis. Since K22 apparently interferes with these processes, inhibitors like K22 and corresponding escape mutants will likely become valuable tools to further our understanding on the induction of membrane alterations and DMV formation that take place during the early phase of the coronavirus life cycle. For example, co-expression of nsp3, nsp4 and native or mutated nsp6 in the absence of virus replication, similar as described by Angelini and colleagues [39], may help to clarify whether presence of K22 would affect formation of DMV by directly targeting nsp6 or cellular protein(s) required and recruited for DMV formation.

We emphasize that the identification of K22 and its proposed mode-of-action is only the very first step towards an approved drug for therapeutic use in animals or humans. Specifically, we are currently focusing on the structure-activity relationship analysis of K22 analogs, with the aim to identify compounds with improved antiviral and cytotoxic profiles prior to their assessment *in vivo*. However, one important lesson of the past SARS-CoV and recent MERS-CoV outbreaks is that zoonotic transmission of coronaviruses into the human population can pose considerable threat to human health and that it is warranted to eventually invest significant efforts to developing efficacious and approved drugs to increase preparedness and combat coronavirus infections. The antiviral activity against a number of diverse coronaviruses makes K22 an ideal candidate for further development towards an efficacious “pan-coronavirus inhibitor”. Broad anti-coronaviral activity has been proposed for inhibitors targeting highly conserved enzymatic functions, such as coronavirus proteinase activities [26], [58], or more recently, for compounds targeting host cell factors required for efficient replication, such as cyclophilins [59], [60]. The concept of targeting multiple key functions of viral replication led to the development of efficacious treatment regimens against HIV and hepatitis C virus by combining multiple antiviral drugs [61], [62] and it is tempting to speculate that this concept will be applicable to combat coronavirus infections in the future. Moreover, with the identification of K22, we demonstrate that there are yet additional critical steps in the life cycle of positive strand RNA viruses to explore as targets for antiviral intervention.

## Materials and Methods

### Ethics statement

Human bronchial epithelial cells were isolated from patients (>18 years old) who underwent bronchoscopy and/or surgical lung resection in their diagnostic pathway for any pulmonary disease and that gave written informed consent. This was done in accordance with local regulation of the Kanton St. Gallen, Switzerland, as part of the St. Gallen Lung Biopsy Biobank (SGLBB) of the Kantonal Hospital, St. Gallen, which received approval by the ethics committee of the Kanton St. Gallen (EKSG 11/044, EKSG 11/103).

### Cells and viruses

Human embryonic lung diploid fibroblasts (MRC-5), African green monkey kidney cells (Vero), baby hamster kidney cells (BHK-21), felis catus whole fetus 4 cells (FCWF-4), were purchased from the American Type Culture Collection (ATCC), murine fibroblast cells (L929), African green monkey kidney cells (CV-1) were purchased from the European Collection of Cell Cultures. D980R cells were a kind gift from G. L. Smith, Imperial College, London, United Kingdom. African green monkey kidney (GMK AH1) cells were obtained from the Swedish Institute for Infectious Disease Control, Stockholm. Cells were grown in Eagle's minimum essential medium (EMEM) (MRC-5, CV-1, D980R, L929, BHK-21, GMK AH1 cells) or in Dulbecco's modified EMEM (DMEM) (FCWF-4, Vero cells), supplemented with 5–10% heat-inactivated fetal calf serum, (HI-FCS), 1% L-glutamine, penicillin (60 µg/ml) and streptomycin (100 µg/ml) (PEST). Isolation and cultivation of primary human bronchial epithelial cells to form pseudostratified/differentiated human airway epithelial (HAE) cultures was performed as described previously [15], [63].

Human CoV strain 229E [4] (HCoV-229E) was obtained from ATCC (VR-740). HCoV-229E stocks were prepared from virus passages 6–8 in MRC-5 cells growing in EMEM supplemented with 2% HI-FCS, 1% L-glutamine, HEPES (10 mM) and PEST (EMEM-FP). In some experiments, the virus was concentrated by centrifugation of infectious culture fluid of MRC-5 cells over a 1.5 ml cushion of 20% sucrose for 2 h at 22000 rpm (SW28.1 rotor, Beckman). The pellet was covered with PBS (137 mM NaCl, 2.7 mM KCl, 8.1 mM Na2HPO4, 1.5 mM KH2PO), left overnight at 4°C, and then gently suspended by pipetting. The following viruses and their propagation were described previously: recombinant HCoV- 229E [64], recombinant HCoV-229E-Ren expressing Renilla luciferase [52], recombinant feline coronavirus (strain Black) expressing Renilla luciferase (recFCoV-RL) [48], SARS-CoV strain Frankfurt-1 [50], recombinant avian infectious bronchitis virus (IBV, strain Beaudette) [49], MERS-CoV [8], [51]. Recombinant MHV strain A59 expressing Gaussia luciferase (MHV-Gluc) was generated based on the previously described reverse genetics system [47], [65]. Briefly, the MHV-A59 accessory gene 4 was replaced by the gene encoding the codon-optimized Gaussia luciferase [66] (hGLuc) using vaccinia-virus-mediated homologous recombination essentially as described for the generation of MHV-GP33-GFP [67]. The plasmid DNA used for recombination contained MHV-A59 nucleotides (nts) 27500–27967, the hGLuc Gaussia luciferase gene, and MHV-A59 nts 28265–28700. Recombinant HCoV-229E containing mutations conferring K22 resistance in nsp6 were generated based on the previously described reverse genetics system [64], [65]. Briefly, vaccinia virus HCoV-inf1 (containing the full-length HCoV-229E cDNA) [64] was used to recombine with a plasmid based on pGPT1 [68] where the *Escherichia coli* guanine phosphoribosyltransferase (GPT) gene was flanked by HCoV-229E nts 9398–10098 and 10930–11580. The resulting GPT-positive vaccinia virus was then used to recombine with plasmids containing the HCoV-229E nts 9398–11580 with modification of nucleotide 10455 (A to T; HCoV-229E^H121L^), or nt 10568 (A to G; HCoV-229E^M159V^), or both nts 10455 and 10568 (HCoV-229E^H121L/M159V^). The resulting vaccinia viruses were then used to rescue HCoV-229E^H121L^, HCoV-229E^M159V^, and HCoV-229E^H121L/M159V^ as described previously [64], [65]. The identity of plasmid DNA and recombinant vaccinia viruses and recombinant coronaviruses was confirmed by sequencing. In some experiments poliovirus 1 strain Sabin (obtained from the Swedish Institute for Infectious Disease Control, Stockholm) was used.

### Reagents

The ChemBioNet diversity library of 16671 compounds was obtained from the Leibniz Institute for Molecular Pharmacology (Berlin, Germany). Library was provided in a 384 well plate format, each well containing 5 µl of a compound solubilized in DMSO to a final concentration of 10 mM. Hit compound K22 was purchased from ChemDiv (San Diego, CA; catalog number 4295–0370). The correct structure and purity of K22 (>95%) was confirmed in our laboratory by NMR and LCMS analyses.

### Immunofluorescence analysis

MRC-5 cells were infected at a multiplicity of infection (moi) of 0.05 with wtHCoV-229E and K22-resistant recombinants HCoV-229E^H121L^, HCoV-229E^M159V^, and HCoV-229E^H121L/M159V^ with or without the presence of K22 (4 µM). The cells were fixed at 18 h p.i. with 4% paraformaldehyde (PFA) and immunostained [69] using the mouse monoclonal anti-dsRNA (J2, English & Scientific Consulting Bt.) and rabbit anti-HCoV-229E nsp8 [70] (kindly provided by John Ziebuhr, University of Giessen, Germany) as primary antibodies for detection of double-stranded (ds) RNA and viral replication complexes. Donkey derived, Dylight 488 labeled, anti-mouse IgG (H+L) and Dylight 647 labeled, anti-rabbit IgG (H+L) (Jackson Immunoresearch) were applied as secondary antibodies. Cells were counterstained with DAPI (4',6-diamidino-2-phenylindole; Invitrogen) to visualize nuclei. HAE cell cultures were inoculated with 40000 plaque forming units (PFU), with or without the presence of K22 (50 µM) and fixed with 4% PFA 48 h p.i. Staining was performed with the mouse monoclonal antibody directed against dsRNA (J2) and goat polyclonal anti-ZO1 (tight junctions; ab99462, Abcam) as primary antibodies. Dylight 488-labeled donkey anti-mouse IgG (H+L), Dylight 546-labeled donkey anti-goat IgG (H+L) (Jackson Immunoresearch) were applied as secondary antibodies, followed by two separate incubation steps with Alexa Fluor647-conjugated rabbit monoclonal anti-beta-Tubulin antibody (ciliated cells; 9F3, Cell Signal) and DAPI (Invitrogen). Images were acquired using EC-plan Neofluar 20x/50 M27 or EC Plan-Neofluar 40x/1.30 Oil DIC M27 objectives on a Zeiss LSM 710 confocal microscope. Image capture, analysis and processing were performed using the ZEN 2010 (Zeiss) and Imaris (Bitplane Scientific Software) software packages.

### Anti-coronavirus compound screening assay

The screening assay was performed as described previously for respiratory syncytial virus [71]. Briefly, MRC-5 cells were seeded in 384 well plates (CLS-3701; Costar-Corning, NY, USA) to become ∼70–90% confluent after one day of culture. The growth medium was removed, and the cells supplemented consecutively with 25 µl of EMEM-FP medium, 1 µl volumes of library compounds at 1 mM concentration, and ∼350 PFU of HCoV-229E in 25 µl of EMEM-FP. The last two columns of the 384 well plate received either virus or EMEM-FP medium to serve as controls. The cells were observed under the microscope for their protection from the virus-induced cytopathic effect after 3 and 6 days of incubation at 37°C.

### Antiviral assays

Plaque reduction assay to determine the antiviral effect of K22 on HCoV-229E was done as follows. MRC-5 cells were seeded in 12-well plates to become nearly confluent after one day of culture. Serial fivefold dilution of K22 (0–100 µM) and 100 PFU of HCoV-229E virus in 0.5 ml of EMEM-FP medium were added to and incubated with cells for 3 h at 37°C, 5% CO2. Subsequently, the virus-compound mixtures were removed from cells, and 1.5 ml volumes of 1% methylcellulose (MC) solution in EMEM-FP medium supplemented with the same concentration of K22 were added. The plates with cells were further incubated at 37°C, 5% CO2 for 2–3 days, and then stained with 0.2% solution of crystal violet to visualize the viral plaques.

Viral yield reduction assays were done to determine the antiviral effect of K22 on HCoV-229E-Ren, recFCoV-RL, MHV-Gluc, SARS-CoV, IBV, MERS-CoV, and poliovirus replication. Briefly, K22 or its DMSO solvent in medium was added at the indicated concentrations to nearly confluent monolayers of corresponding cell lines or to HAE cultures at the basolateral side and incubated for 4 h at 37°C, 5% CO2. The cells were then inoculated with recFCoV-RL (moi = 0.1 on FCWF-4 cells), MHV-Gluc (moi = 0.001 on L929 cells), SARS-CoV (moi = 0.001 on Vero cells), IBV (moi = 1 on Vero cells), HCoV-229E-Ren (4×10^3^ PFU on HAE cultures apically), MERS-CoV (4×10^3^ PFU on HAE cultures apically) or poliovirus (moi = 0.001 on GMK AH1 cells). After 2 h the viral inoculum was removed, cells were rinsed three times with PBS, and fresh medium containing the same concentrations of K22 or DMSO was added. Coronavirus replication was assessed from cell culture supernatant by determining titer as TCID50 (tissue culture infectious dose that will produce pathological change in 50% of cell cultures inoculated) for IBV or poliovirus at 48 h p.i., by determining the amount of viral genome RNA produced by qRT-PCR specific for SARS-CoV and MERS-CoV at 48 h p.i. as described previously [51], or by determining the level of Renilla expression at 48 h p.i. (HCoV-229E-Ren) or 72 h p.i. (recFCoV-RL) using *Renilla* Luciferase Assay System (Promega, E2820), or Gaussia luciferase expression (MHV-Gluc) at 24 h p.i. using the BioLux *Gaussia* Luciferase Assay Kit (NEB,E3300), respectively.

For the virucidal assay, 200 µl of HCoV-229E suspension (∼3×10^4^ PFU) in EMEM-FP medium was mixed with 50 µM K22 and incubated for 15 min at 37°C. In the control sample, virus was incubated with the DMSO solvent at a final concentration corresponding to that present in the test compound. Then, both mixtures were diluted serially tenfold in EMEM-FP medium and the residual virus infectivity determined by the viral plaque assay.

### Cell toxicity and proliferation assays

The toxicity of K22 or its solvent (DMSO) for MRC-5 cells was evaluated using the tetrazolium-based CellTiter 96 AQueous One Solution cytotoxicity assay (Promega; G3580). The effect of K22 or its solvent on proliferation of MRC-5 cells was studied as follows. The cells were seeded in 48 well plates to become ∼50% confluent after one day of culture. The growth medium was removed, and cells incubated with specific concentrations of K22 or its solvent in EMEM-FP medium for 72 h at 37°C. The cells were then dissociated with trypsin/EDTA solution and counted. The effect of K22 or DMSO on viability of Vero, L929, and FCFW-4 cells was assessed using the CytoTox-Glo Cytotoxicity Assay kit (Promega, G9291) while the toxicity of test compound for differentiated HAE cultures was evaluated with CellTiter-Glo Luminescent Cell Viability Assay kit (Promega, G7571).

### Time-of-addition assay

MRC-5 cells growing in 12 well plates were precooled for 15 min at room temperature and for another 15 min at 4°C. The cells were rinsed once with 500 µl of cold EMEM-FP and inoculated with HCoV-229E at moi of 0.05. Following virus adsorption to cells for 45 min at 4°C, the cells were rinsed twice with 500 µl of cold EMEM-FP, and 990 µl of warm EMEM-FP medium was added. Subsequently 10 µl of 1 mM K22 was added at specific time points relative to the end of the virus adsorption period, and the infectious cell culture medium and cells harvested at the time point 24 h. The cell culture supernatant medium was clarified by centrifugation at 1000×*g* for 5 min while the pelleted cells were suspended in RNase-free water and stored at −80°C until quantification in RT-PCR assay. To study the effect of K22 on early virus-cell interaction the “time-of-addition” assay was modified as follows. MRC-5 cells were rinsed once with 1 ml of EMEM-FP and 500 µl of EMEM-FP supplemented with 4 µM K22 was added. The compound was incubated with cells for 2 h at 37°C either prior to, during or after a 2 h period of infection of cells with ∼100 PFU of 229E virus in 500 µl of EMEM-FP. The cells were washed once with 1 ml of EMEM-FP after each 2 h period of their incubation with compound and/or virus. Finally, the cells were overlaid with the MC solution, and after incubation for 2 days at 37°C stained with crystal violet to visualize the viral plaques.

### RT-PCR

The RT TaqMan PCR was carried out as described by Brittain-Long et al. [72]. Briefly, the extraction of RNA was conducted in the Magnapure LC robot using the MagNA Pure LC Total Nucleic Acid Isolation Kit (Roche Applied Science, Mannheim, Germany), and amplification was performed using a TaqMan 7300 Real Time PCR system (Applied Biosystems, Foster City, CA), with a pair of forward 5′-CAGTCAAATGGGCTGATGCA-3′ and reverse 5′-AAAGGGCTATAAAGAGAATAAGGTATTCT-3′ primers as well as a probe 3′CCCTGACGACCACGTTGTGGTTCA 5′ specific for HCoV-229E genome fragment coding for nucleocapsid protein [73]. The number of HCoV-229E RNA copies was determined by relating the detected cycle threshold values to a standard curve prepared based on five tenfold dilutions of the specific plasmid (pUC57) comprising a 94 bp insert from the nucleocapsid sequence of HCoV-229E. qRT-PCR assays to quantify SARS-CoV and MERS-CoV genomic RNA have been described previously [51].

### Preparation of drug-resistant variants of HCoV-229E and sequencing analysis

A procedure described previously for respiratory syncytial virus [71] was used. Briefly, plaque purified HCoV-229E was subjected to 10–13 consecutive passages in MRC-5 cells in the presence of increasing concentrations (2–16 µM) of K22. For control purposes, the same virus was also passaged in MRC-5 cells in the absence of inhibitor. The virus was then subjected to two rounds of plaque purification in the presence of inhibitor, and its relative drug-resistance tested using the viral plaque reduction assay. Genomic RNA of original, mock-passaged, and the K22-resistant virus from passage 10–13 was extracted from extracellular fluid of the 229E-infected MRC-5 cells using the QIAamp viral RNA purification kit (Qiagen). Overlapping DNA fragments covering the entire coding sequence were produced by reverse transcription PCR and subjected to nucleotide sequencing using the ABI PRISM Big Dye Terminator v3.1 Cycle Sequencing Ready Reaction kit (Applied Biosystems). Nucleotide sequence analysis was performed using Sequencher 4.9 software (Gene Codes Corporation).

### HCoV-229E replication kinetics

MRC-5 cells growing in 12 well plates were precooled for 15 min at room temperature and for another 15 min at 4°C. The cells were rinsed once with 500 µl of cold EMEM-FP and inoculated with concentrated preparation (see the Cells and Viruses section) of HCoV-229E (moi = 0.05). Following virus adsorption to cells for 1 h at 4°C, the cells were rinsed thrice with 500 µl of cold EMEM-FP, and 500 µl of warm EMEM-FP medium was added. The supernatant fluid and infected cells were harvested at specific time points relative to the end of the virus adsorption period, and processed for determination of viral RNA and infectivity as described under the “time-of-addition” assay.

### Ribonuclease treatment of HCoV-229E

The infectious culture medium comprising HCoV-229E or recombinant nsp6 mutant HCoV-229E M159V were clarified by centrifugation at 1000×g for 5 min, and then 100 µl volumes of the supernatant were supplemented with 2 µl (20 µg) of ribonuclease A (Thermo Fisher Scientific; EN0531) or its solvent. All samples were spiked with ∼7 µg of RNA purified from human respiratory syncytial virus (RSV) to serve as an internal control of ribonuclease activity. Following incubation of the virus-enzyme mixture for 30 min at 37°C, the coronaviral and RSV RNA were quantified by RT TaqMan PCR as described by Brittain-Long et al. [72] while coronavirus infectivity was determined by plaque titration.

### Autophagy

To assess the time-frame where autophagy vesicle formation occurs we seeded Huh-7 cells (100.000 cells) on glass bottom 12-well cluster plates (MatTek). Forty-eight hours prior to stimulation cells were transfected with LC3B-GFP plasmid [74] using lipofectamine2000 (Invitrogen), according to manufactures protocol. Hereafter cells were exposed to 100 nM of rapamycin (Invivogen) alone or in presence of either 20 µM of K22 or an equal volume of DMSO for the duration of 18 hours at 37°C. Fluorescent and differential interference contrast (DIC) images were acquired with 30 minute interval using EC Plan Neo-fluar 40x/1.30 Oil DIC M27 objective on a Zeiss LSM 710 confocal microscope. Image capture, analysis and processing were performed using the ZEN 2010 (Zeiss). To determine whether K22 inhibits endogenous autophagy vesicle formation we stimulated Huh-7 cells (40.000 cells) with 100 nM of rapamycin alone or in presence of either 20 µM of K22 or an equal volume of DMSO for duration of six hours at 37°C. Unstimulated cells were used as mock control. Cells were fixed and immunostained as previously described [69]. Rabbit polyclonal anit-LC3B (L7543, Sigma Aldrich) was applied as primary antibody for the detection of autophagy vesicles. Goat derived, Cy3 labeled, anti-rabbit IgG (H+L; Jackson ImmunoResearch) was applied as secondary antibody. Thereafter cells were counterstained with DAPI (Invitrogen). Fluorescent images were acquired using a PLAPON 60xO/1.42 objective on an Olympus FV-1000 confocal microscope. Image capture, analysis and processing were performed using the Olympus Fluoview software.

### Electron microscopy

MRC-5 cells growing on a Melinex polyester film (Agar Scientific Ltd., Stansted, U.K.) in 24 well cluster plates were infected with HCoV-229E (moi = 0.04) in the presence of 10 µM of K22. After 18 h of infection at 37°C, the culture medium was removed, the cells rinsed twice with Eagle's medium, and a fresh Eagle's medium supplemented with 2.5% glutaraldehyde was added and incubated for 45 min at 37°C. The cells were washed twice with 0.05 M Tris-HCl buffer (pH 7.4) supplemented with 2 mM CaCl2, and further processed for electron microscopy as described [75]. Experiments with recombinant nsp6 mutant viruses and original virus were carried out in a similar manner except that the cells were inoculated at a moi of ∼0.25 and incubated with or without the presence of 4 µM K22.

## Supporting Information

Figure S1 **J15 structure, antiviral activity, and cytotoxicity.** (**A**) J15 structure. (**B**) Anti-HCoV-229E activity and cytotoxicity of J15 in MRC-5 cells. J15 and wild type (WT) HCoV-229E or nsp6 recombinant HCoV-229E^M159V^ (M159V) were added to MRC-5 cells, and the number of viral plaques developed after 48 h were assessed. For cytotoxicity assessment, MRC-5 cells were incubated with J15 for 48 h at 37°C and the cell viability determined using tetrazolium-based reagent. Data shown are means (±SD) of duplicate determinations from two independent experiments. PFU, plaque forming unit.
(TIF)

Figure S2 **Ribonuclease treatment of HCoV-229E.** Infectious culture medium comprising wild type HCoV-229E or mutant nsp6 recombinant HCoV-229E^M159V^ (M159V) was spiked with RNA purified from human respiratory syncytial virus (RSV) and then incubated for 30 min at 37°C in the presence of ribonuclease A (RNase) or without this enzyme (mock). The number of copies of coronaviral RNA (A) or control RSV RNA (B) was determined by qPCR while titer of infectious coronavirus (C) by viral plaque assay. Data shown are means (±SD) of four determinations obtained in four independent experiments (qPCR) or duplicate determinations from two independent experiments (infectivity). PFU, plaque forming unit; n.d., not detectable; n.s., not significant.
(TIF)

Figure S3 **J15 affects formation of double membrane vesicles (DMVs).** MRC-5 cells growing on Melinex polyester film were infected with wild type HCoV-229E (WT) or with K22-resistant recombinant nsp6 mutant HCoV-229E^M159V^ (M159V) and incubated for 18 h at 37°C with or without J15. The cells were then fixed with glutaraldehyde and processed for electron microscopy without their scrapping or pelleting. (**A**) Electron micrographs of cells infected with WT virus show presence of clusters of DMVs (arrow) and viral particles (arrowhead), and the lack of their production upon J15 treatment (4 µM). (**B**) Electron micrographs of MRC-5 cells infected with K22-resistant recombinant nsp6 mutant M159V showing presence of DMVs and viral particles irrespective of the addition of J15.
(TIF)

Figure S4 **K22 does not inhibit autophagy vesicle formation.** To determine whether K22 inhibits autophagy vesicle formation Huh-7 cells were stimulated with rapamycin alone or in presence of either 20 µM of K22 or an equal volume of DMSO solvent for 6 h at 37°C. Unstimulated cells were used as mock control. Fixed cells were stained with Anti-LC3B (red) and DAPI (blue) to annotate autophagy vesicles and cell nucleus, respectively.
(TIF)

Figure S5 **K22 affects replication of diverse coronaviruses including MERS-CoV.** (**A-D**) The antiviral activity (bars) and cell toxicity (data points above bars) of K22 (black bars) or DMSO solvent (white bars) during MHV-Gluc (**A**), FCoV-RL (**B**), SARS-CoV (**C**) and IBV (**D**) infection on representative continuous cell lines of murine (L-929 cells; **A**), feline (FCWF cells; **B**), or primate (Vero cells; **C-D**) origin. Data are shown as mean (±SD) of a representative experiment, from two independent experiments performed in triplicate. (**E-F**). The antiviral activity (bars) and cell toxicity (data points above bars) of K22 (black bars) or DMSO solvent (white bars) in HCoV-229E-ren (**E**) and MERS-CoV (**F**) infected differentiated human airway epithelial (HAE) cultures. Data are shown as mean (±SD) of three independent experiments performed in triplicate (viral yield), or mean (±SD) of a representative experiment, from two independent experiments performed in triplicate (cell viability). Ns, not significant (*P*>0.05); * *P*<0.05; ** *P*<0.01 (paired t-test).
(TIF)

Figure S6 **K22 exhibits little or no activity against poliovirus 1.** GMK AH1 cells were pretreated with K22 (black bars) or DMSO solvent (white bars) for 4 h at 37°C and then infected with poliovirus 1 Sabin strain at a moi of 0.001. Following incubation of infected cells in the presence of K22 or DMSO for 48 h at 37°C, the titer of extracellular infectious virus in culture medium was determined. The results shown are means of duplicate determinations from two separate experiments. TCID_50_, tissue culture infectious dose.
(TIF)

Table S1 **Effect of K22 on proliferation and viability of cultured cells.**
(DOCX)
