# Supplementary figures and images for: Targeting Membrane-Bound Viral RNA Synthesis Reveals Potent Inhibition of Diverse Coronaviruses Including the Middle East Respiratory Syndrome Virus
Source: PLoS Pathog. 2014 May 29;10(5):e1004166. doi: 10.1371/journal.ppat.1004166 (PMC4038610; doi:10.1371/journal.ppat.1004166)

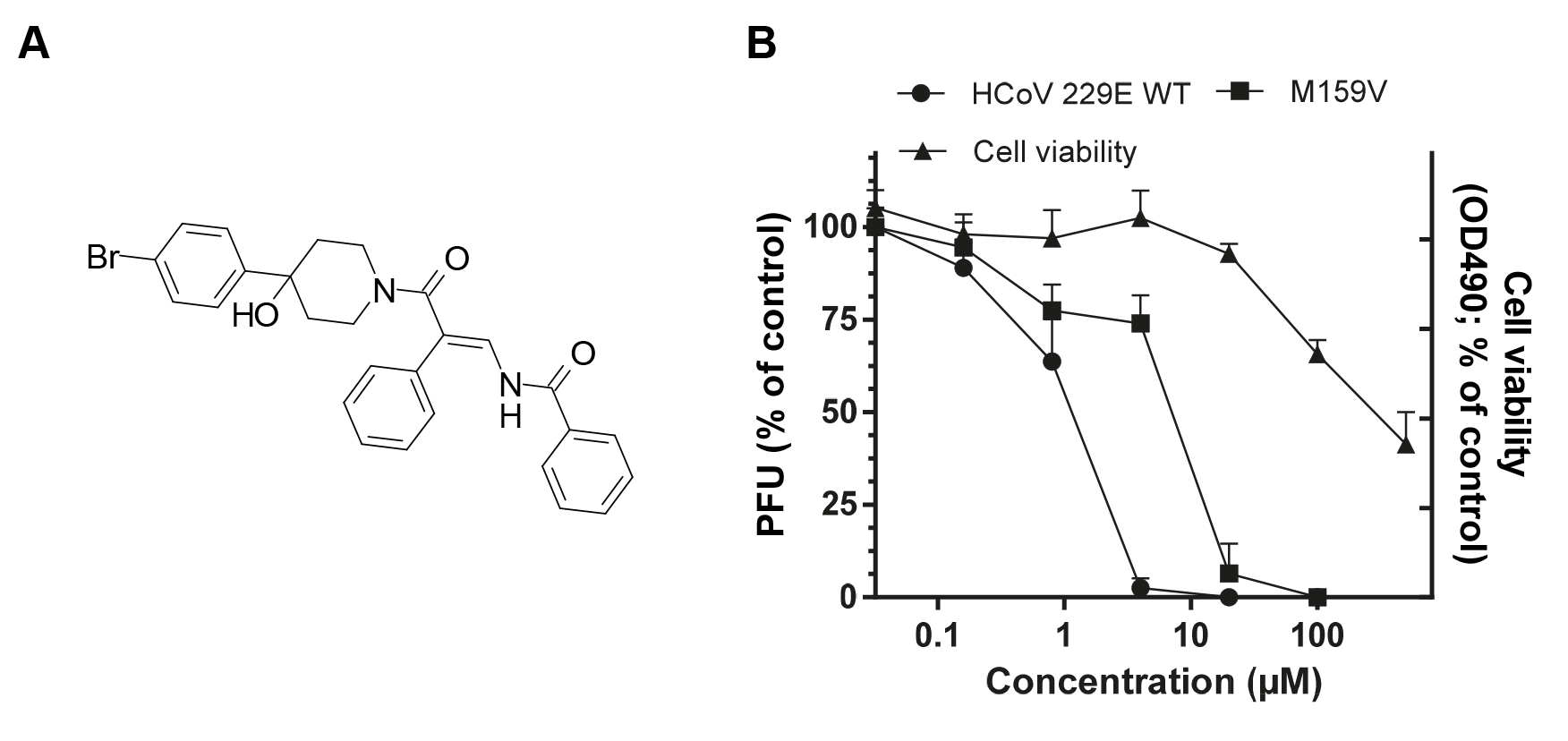

Supplement: Figure S1 — J15 structure, antiviral activity, and cytotoxicity. (A) J15 structure. (B) Anti-HCoV-229E activity and cytotoxicity of J15 in MRC-5 cells. J15 and wild type (WT) HCoV-229E or nsp6 recombinant HCoV-229EM159V (M159V) were added to MRC-5 cells, and the number of viral plaques developed after 48 h were assessed. For cytotoxicity assessment, MRC-5 cells were incubated with J15 for 48 h at 37°C and the cell viability determined using tetrazolium-based reagent. Data shown are means (±SD) of duplicate determinations from two independent experiments. PFU, plaque forming unit. (TIF) [file ppat.1004166.s001.tif]

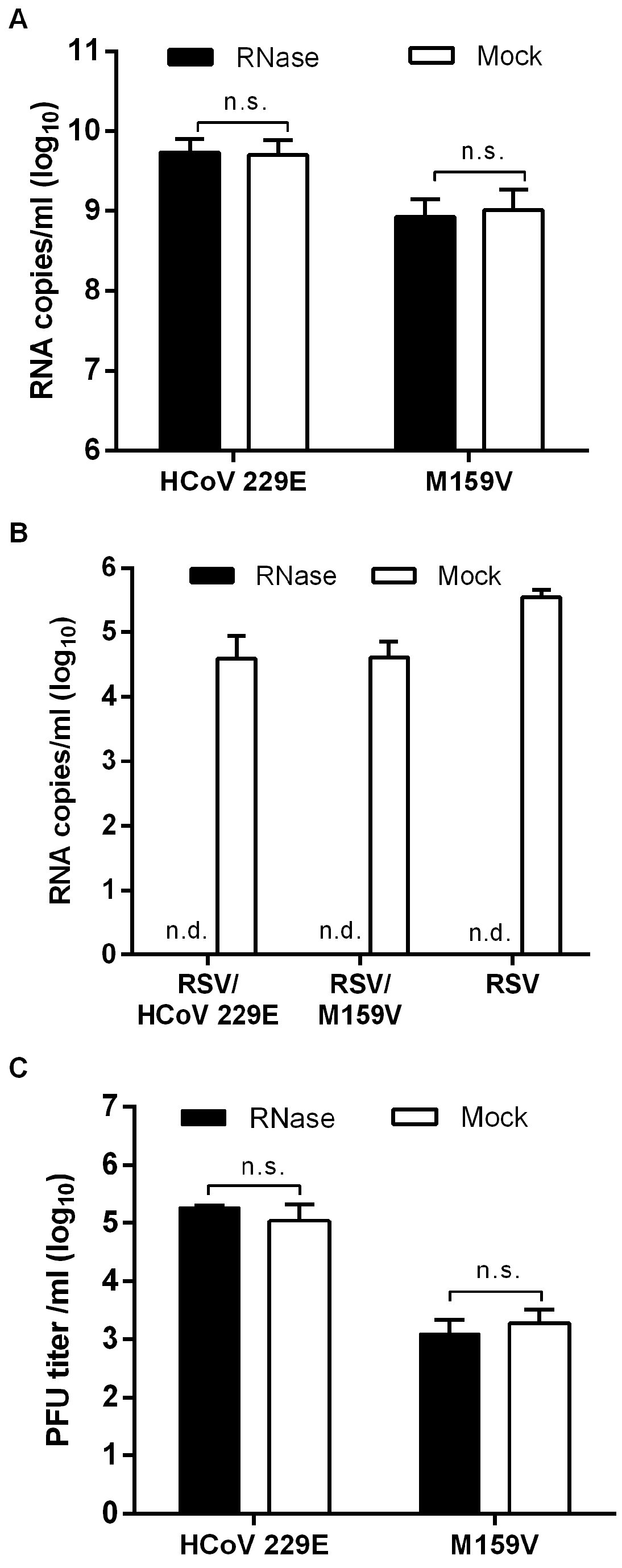

Supplement: Figure S2 — Ribonuclease treatment of HCoV-229E. Infectious culture medium comprising wild type HCoV-229E or mutant nsp6 recombinant HCoV-229EM159V (M159V) was spiked with RNA purified from human respiratory syncytial virus (RSV) and then incubated for 30 min at 37°C in the presence of ribonuclease A (RNase) or without this enzyme (mock). The number of copies of coronaviral RNA (A) or control RSV RNA (B) was determined by qPCR while titer of infectious coronavirus (C) by viral plaque assay. Data shown are means (±SD) of four determinations obtained in four independent experiments (qPCR) or duplicate determinations from two independent experiments (infectivity). PFU, plaque forming unit; n.d., not detectable; n.s., not significant. (TIF) [file ppat.1004166.s002.tif]

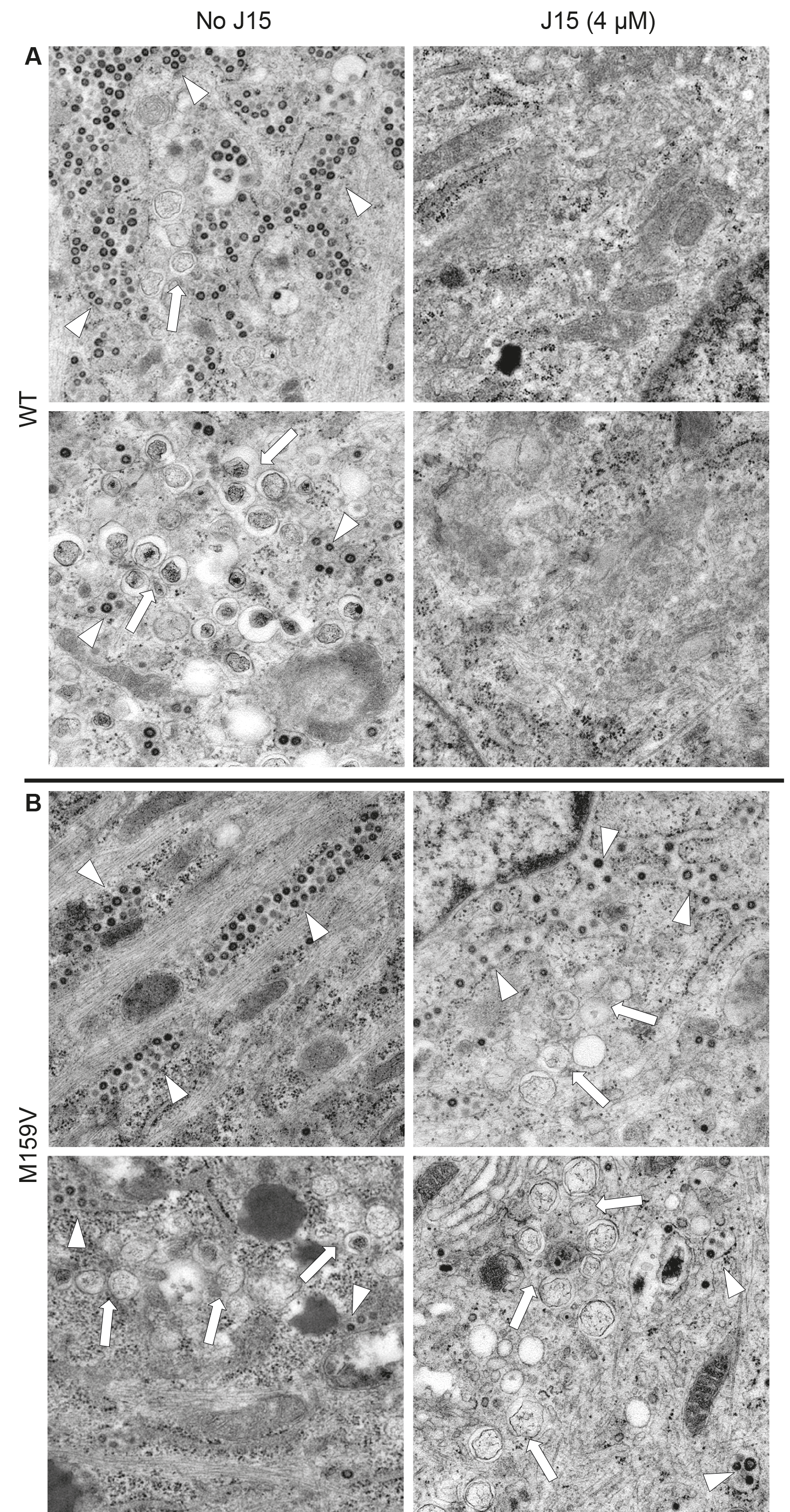

Supplement: Figure S3 — J15 affects formation of double membrane vesicles (DMVs). MRC-5 cells growing on Melinex polyester film were infected with wild type HCoV-229E (WT) or with K22-resistant recombinant nsp6 mutant HCoV-229EM159V (M159V) and incubated for 18 h at 37°C with or without J15. The cells were then fixed with glutaraldehyde and processed for electron microscopy without their scrapping or pelleting. (A) Electron micrographs of cells infected with WT virus show presence of clusters of DMVs (arrow) and viral particles (arrowhead), and the lack of their production upon J15 treatment (4 µM). (B) Electron micrographs of MRC-5 cells infected with K22-resistant recombinant nsp6 mutant M159V showing presence of DMVs and viral particles irrespective of the addition of J15. (TIF) [file ppat.1004166.s003.tif]

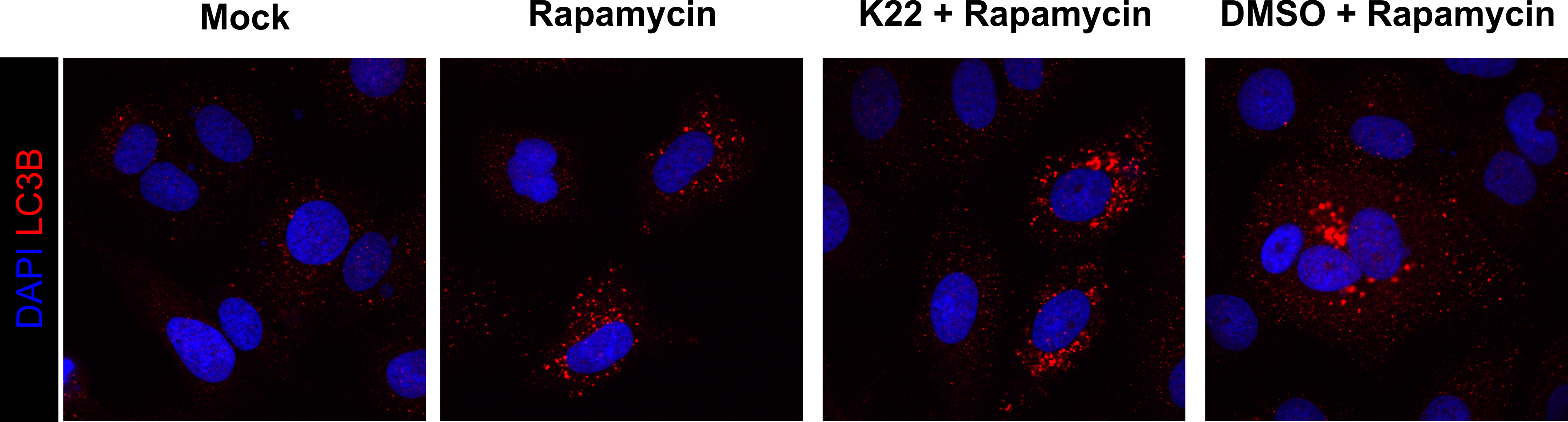

Supplement: Figure S4 — K22 does not inhibit autophagy vesicle formation. To determine whether K22 inhibits autophagy vesicle formation Huh-7 cells were stimulated with rapamycin alone or in presence of either 20 µM of K22 or an equal volume of DMSO solvent for 6 h at 37°C. Unstimulated cells were used as mock control. Fixed cells were stained with Anti-LC3B (red) and DAPI (blue) to annotate autophagy vesicles and cell nucleus, respectively. (TIF) [file ppat.1004166.s004.tif]

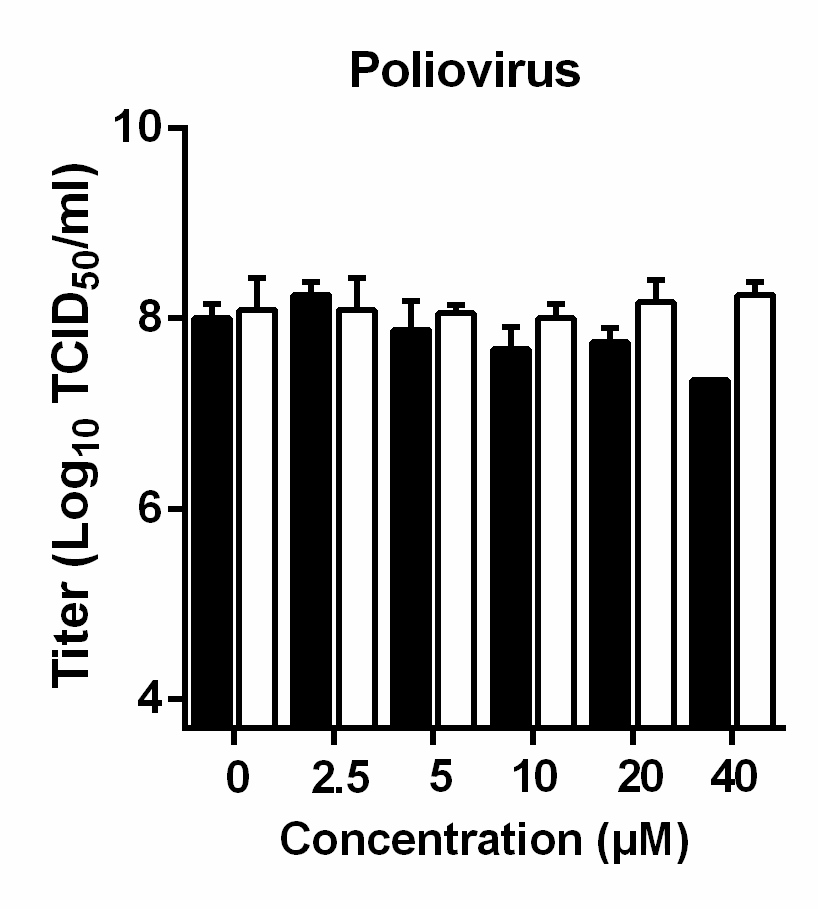

Supplement: Figure S6 — K22 exhibits little or no activity against poliovirus 1. GMK AH1 cells were pretreated with K22 (black bars) or DMSO solvent (white bars) for 4 h at 37°C and then infected with poliovirus 1 Sabin strain at a moi of 0.001. Following incubation of infected cells in the presence of K22 or DMSO for 48 h at 37°C, the titer of extracellular infectious virus in culture medium was determined. The results shown are means of duplicate determinations from two separate experiments. TCID50, tissue culture infectious dose. (TIF) [file ppat.1004166.s006.tif]
