# Supplementary material for: Targeting Membrane-Bound Viral RNA Synthesis Reveals Potent Inhibition of Diverse Coronaviruses Including the Middle East Respiratory Syndrome Virus
Source: PLoS Pathog. 2014 May 29;10(5):e1004166. doi: 10.1371/journal.ppat.1004166 (PMC4038610; doi:10.1371/journal.ppat.1004166)
Supplement: Table S1 — Effect of K22 on proliferation and viability of cultured cells. (DOCX) [file ppat.1004166.s007.docx]

**Supporting Table S1**

**Table S1**

**Effect of K22 on proliferation and viability of cultured cells**

| Cells | Cytostatic activity of K22 (CC_50_; µM)^a^ | Cytotoxic activity of K22 (CC_50_; µM)^b^ |
| --- | --- | --- |
| Vero | 48 | >40 |
| L929 | 57 | >40 |
| FCWF | 40 | >40 |

^a^ Assessed in cell proliferation assay

^b^ Assessed using CytoTox-GloTM cytotoxicity assay kit (Promega, G9291)
